# Development of Immortalized Human Tumor Endothelial Cells from Renal Cancer

**DOI:** 10.3390/ijms20184595

**Published:** 2019-09-17

**Authors:** Nako Maishi, Hiroshi Kikuchi, Masumi Sato, Hiroko Nagao-Kitamoto, Dorcas A. Annan, Shogo Baba, Takayuki Hojo, Misa Yanagiya, Yusuke Ohba, Genichiro Ishii, Kenkichi Masutomi, Nobuo Shinohara, Yasuhiro Hida, Kyoko Hida

**Affiliations:** 1 Vascular Biology and Molecular Pathology, Hokkaido University Graduate School of Dental Medicine, Sapporo 060-8586, Japan; mnako@den.hokudai.ac.jp (N.M.); annandorcasam@gmail.com (D.A.A.); s.baba.0508@gmail.com (S.B.); 2 Vascular Biology, Frontier Research Unit, Institute for Genetic Medicine, Hokkaido University, Sapporo 060-0815, Japan; hiroshikikuchi16@yahoo.co.jp (H.K.); masumi.sato0907hu@alumni.tus.ac.jp (M.S.); ta-hojo@den.hokudai.ac.jp (T.H.); miya1031@den.hokudai.ac.jp (M.Y.); 3 Department of Vascular Biology, Hokkaido University Graduate School of Dental Medicine, Sapporo 060-8586, Japan; hiro.al2o3@gmail.com; 4 Department of Renal and Genitourinary Surgery, Hokkaido University Graduate School of Medicine, Sapporo 060-8638, Japan; nozomis@mbj.nifty.com; 5 Department of Dental Anesthesiology, Hokkaido University Graduate School of Dental Medicine, Sapporo 060-8586, Japan; 6 Department of Oral Diagnosis and Medicine, Hokkaido University Graduate School of Dental Medicine, Sapporo 060-8586, Japan; 7 Department of Cell Physiology, Faculty of Medicine and Graduate School of Medicine, Hokkaido University, Sapporo 060-8638, Japan; yohba@med.hokudai.ac.jp; 8 Division of Pathology, Exploratory Oncology Research and Clinical Trial Center, National Cancer Center, Kashiwa 277-8577, Japan; gishii@east.ncc.go.jp; 9 Division of Cancer Stem Cell, National Cancer Center Research Institute, Tokyo 104-0045, Japan; kmasutom@ncc.go.jp; 10 Department of Cardiovascular and Thoracic Surgery, Hokkaido University Graduate School of Medicine, Sapporo 060-8638, Japan; yhida@med.hokudai.ac.jp

**Keywords:** immortalization, tumor endothelial cells, angiogenesis, renal carcinoma

## Abstract

Tumor angiogenesis research and antiangiogenic drug development make use of cultured endothelial cells (ECs) including the human microvascular ECs among others. However, it has been reported that tumor ECs (TECs) are different from normal ECs (NECs). To functionally validate antiangiogenic drugs, cultured TECs are indispensable tools, but are not commercially available. Primary human TECs are available only in small quantities from surgical specimens and have a short life span in vitro due to their cellular senescence. We established immortalized human TECs (h-imTECs) and their normal counterparts (h-imNECs) by infection with lentivirus producing simian virus 40 large T antigen and human telomerase reverse transcriptase to overcome the replication barriers. These ECs exhibited an extended life span and retained their characteristic endothelial morphology, expression of endothelial marker, and ability of tube formation. Furthermore, h-imTECs showed their specific characteristics as TECs, such as increased proliferation and upregulation of TEC markers. Treatment with bevacizumab, an antiangiogenic drug, dramatically decreased h-imTEC survival, whereas the same treatment failed to alter immortalized NEC survival. Hence, these h-imTECs could be a valuable tool for drug screening to develop novel therapeutic agents specific to TECs or functional biological assays in tumor angiogenesis research.

## 1. Introduction

Vascular endothelial growth factor (VEGF) is one of the primary angiogenic factors secreted by tumor cells to induce angiogenesis and obtain oxygen and nutrients from neovasculature to grow. Blood vessels in tumors are also used in the route by which tumor cells spread to distant organs. Therefore, targeting tumor blood vessels is useful to inhibit both tumor growth and metastasis.

In 2004, bevacizumab, an anti-VEGF drug, was first approved by the U.S. Food and Drug Administration as an angiogenesis inhibitor. Several antiangiogenic drugs have been used in combination with other anticancer drugs and improved clinical outcomes [1]. However, the inhibition of VEGF signaling causes systemic adverse effects, such as hypertension and gastrointestinal perforations [2], as VEGF is also an important molecule for physiologic angiogenesis. Therefore, developing antiangiogenic agents that specifically target tumor blood vessels is desired to avoid severe side effects.

Endothelial cells (ECs) in tumors are considered to be the same as those in normal tissues [3]. However, several research groups, including ours, have reported that tumor ECs (TECs) show a distinct phenotype compared with normal ECs (NECs) [4,5,6]. We have isolated and cultured TECs from tumor xenografts grown in nude mice [6] and human tumors in kidney [7,8] and colon [9] cancer. We found that TECs have chromosomal instabilities [6,7] and show proangiogenic properties [10] with different gene expression profiles compared with NECs [11,12,13,14]. Furthermore, TECs exhibit resistance to antineoplastic drugs including paclitaxel [15,16] and 5-fluorouracil [17], with upregulated expression of multidrug resistance 1 (MDR1) and aldehyde dehydrogenase (ALDH) genes. These TEC-specific characteristics have been confirmed in vivo as well. Novel drugs targeting TEC-specific characteristics could thus be ideal antiangiogenic agents to obtain efficient therapeutic effects without unexpected side effects.

Cell-based screening is commonly used to find novel drug candidates, and involves a comprehensive analysis using thousands of chemical compounds to identify those that are toxic for the targeted cells. It is important to recruit appropriate cell types and prepare a sufficient number of cells. The cells to use in the screening process should represent (reflect) their characteristics in vivo as therapeutic targets. To identify novel chemicals that have inhibitory effects on TECs, we need to use TECs or cells that replicate their specific characteristics. However, the number of isolated TECs obtained from tumor tissues is limited, as TECs exist only in about 2% of tumors [10]. Particularly, in the case of primary human TECs, the volume of the surgical excision specimen is limited to that of the isolate. In addition, primary human cultured cells easily undergo cellular senescence, which is an irreversible cell cycle arrest. Therefore, it is difficult to culture a huge number of primary human TECs.

In this study, we established immortalized human TECs (h-imTECs) and NECs (h-imNECs). Primary human TECs (hTECs) were isolated from renal cell carcinoma (RCC) specimens, and human NECs (hNECs) were isolated from the noncancerous regions of the same patient. These ECs were transfected with lentivirus vectors of simian virus 40 (SV40) large T antigen and human telomerase reverse transcriptase (hTERT) and were characterized.

## 2. Results

### 2.1. Establishment of Immortalized ECs

**Figure 1:**
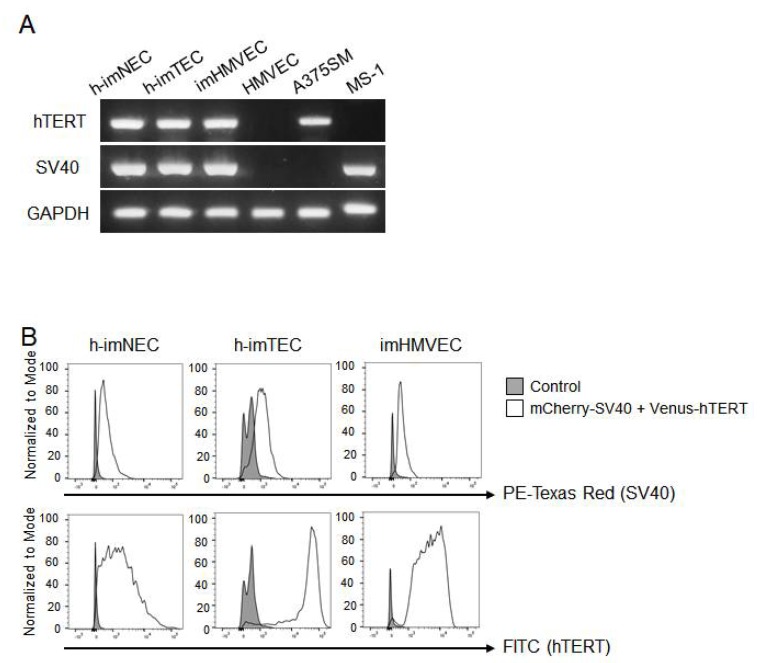
Expression of Venus-hTERT (human telomerase reverse transcriptase) and mCherry-SV40 (simian virus 40) large T antigen in engineered endothelial cells (ECs). (**A**) hTERT and SV40 mRNA expression levels in each EC were determined by RT-PCR. Nontransfected HMVEC (human microvascular endothelial cell) was used as a negative control for hTERT and SV40, and A375SM and MS1 were used as a positive control for hTERT and SV40, respectively. h-imNECs (immortalized human normal endothelial cells) at passage 22 (P22), h-imTECs (immortalized human tumor endothelial cells) (P18), imHMVEC (immortalized HMVEC) (P25), and HMVEC (P9). (**B**) Representative flow cytometric analysis of ECs showing white expression area of mCherry (top) and Venus (bottom). Each nontransfected EC was used as control (gray area).

To generate immortalized ECs, we transducted SV40 large T antigen and hTERT into isolated hTECs and hNECs of renal cancer and human microvascular ECs (HMVECs) by lentiviral infection. The resulting immortalized cells were termed h-imTECs, h-imNECs, and imHMVECs, respectively. The expression of SV40 and hTERT in each EC was analyzed by polymerase chain reaction (PCR). HMVECs without transfection were used as a negative control, and A375SM cells and MS1 cells were used as a positive control for hTERT [18] and SV40 [19], respectively. Both SV40 and hTERT expressions were confirmed in h-imNECs, h-imTECs, and imHMVECs (Figure 1A). To check the purity of transducted ECs, fluorescence signals of mCherry and Venus were analyzed by flow cytometry. As shown in Figure 1B, both mCherry-SV40 large T antigen and Venus-hTERT signals were detected in each transfected EC at high levels.

### 2.2. Extended Life Span of ECs by Ectopic SV40 Large T Antigen and hTERT Expression

**Figure 2:**
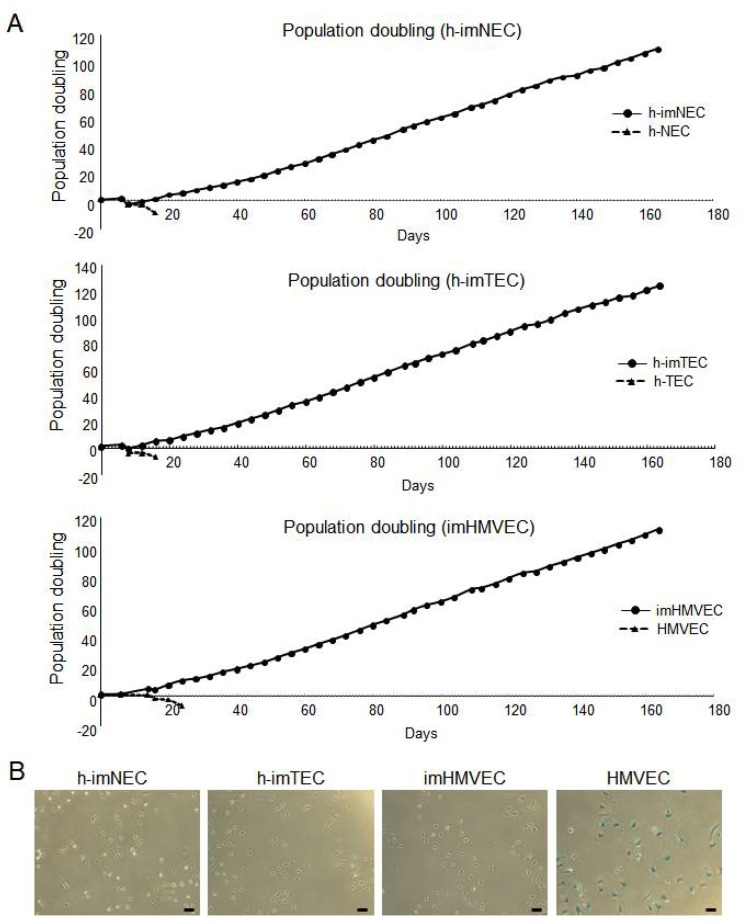
Immortalization of hTERT and SV40 large T antigen transducted ECs. (**A**) Growth kinetics of each EC was measured by cell counting, and population doubling (PD) was calculated. Of note, immortalized ECs continued cell proliferation after 100 to 120 PDs (160 days of culture), whereas all non-immortalized ECs entered the growth arrested state in about 20 days. (**B**) SA-β-Gal staining in each EC. Positive cells had cytoplasmic staining that appeared blue. h- imNECs (P58), h-imTECs (P57), imHMVEC (P57), and HMVEC (P13). Scale bar, 100 μm.

Long-term culture is difficult for human-derived primary cells due to their cellular senescence. To analyze whether SV40 large T antigen and hTERT transduction extended the EC life span, cell proliferation of each EC type was analyzed by cell counting. All non-immortalized ECs entered the growth-arrested state in about 20 days (Figure 2A). In contrast, all immortalized ECs continued cell proliferation after 100 to 120 Population Doubling (PDs) (160 days of culture) (Figure 2A). This is reflected by the expression of SA-β-Gal (Figure 2B). During the aging process, non-immortalized HMVECs (PD16) showed SA-β-Gal activity, whereas h-imNEC (PD160), h-imTEC (PD160), and imHMVECs (PD160) had no SA-β-Gal activity (Figure 2B). These data suggest that SV40 large T antigen and hTERT transduction immortalized ECs.

### 2.3. Maintenance of Endothelial Characteristics in Immortalized ECs

**Figure 3:**
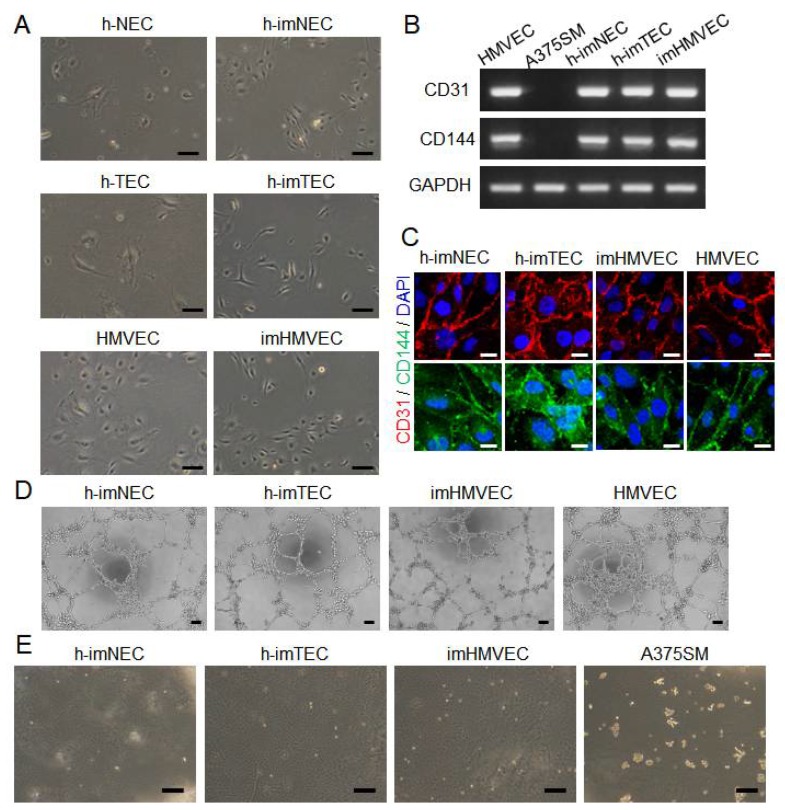
Maintenance of endothelial characteristics in immortalized ECs. (**A**) Representative pictures of immortalized ECs. h-NEC (P11), h-imNEC (P13), h-TEC (P9), h-imTECs (P15), HMVEC (P9), and imHMVEC (P16). Scale bar, 100 μm. (**B**) EC marker, CD31, and CD144 mRNA expression in each EC were determined by RT-PCR. A375SM was used as a negative control. h-imNEC (P22), h-imTECs (P18), imHMVEC(P25), and HMVEC (P9). (**C**) EC marker, CD31, and CD144 protein expression in each EC were determined by immunocytochemistry. h-imNEC (P30), h-imTECs (P31), imHMVEC (P28), and HMVEC (P8). Scale bar, 20 μm. (**D**) Tube formation assay on Matrigel. Scale bar, 100 μm. h-imNEC (P18), h-imTECs (P17), imHMVEC (P18), and HMVEC (P11). (**E**) Soft agar assay. Of note, no colony formation was observed in h-imNEC (P18), h-imTEC (P17), and imHMVEC (P18) in comparison with A375SM (positive control). Scale bar, 200 μm.

We analyzed whether immortalized ECs maintained the characteristics of ECs even after hTERT and SV40 large T antigen transduction. Cells were first observed under the microscope. Representative pictures demonstrated that immortalized ECs did not change their cell shapes after ectopic hTERT and SV40 large T antigen expression (Figure 3A). Second, a EC-specific marker expression was analyzed by PCR and immunocytochemistry. The expression of CD31 (PECAM-1), a pan-endothelial cell marker, was confirmed in all the ECs (Figure 3B,C and Figure S1). One of the transmembrane components of endothelial adherence junction expression, CD144 (VE-cadherin), was also observed in each EC (Figure 3B,C and Figure S1). HMVECs and A375SM, a melanoma cell line, were used as positive and negative controls, respectively. Furthermore, the ability of tube formation on Matrigel was analyzed by tube formation assay. Each immortalized EC type formed tubes (Figure 3D). To examine the tumorigenic potential after hTERT and SV40 large T antigen transduction, the soft agar assay was performed. No colony formation was observed in h-imNECs, h-imTECs, and imHMVECs in comparison with A375SM, which served as a positive control (Figure 3E). These data suggested that immortalized ECs could not grow in an anchorage-independent manner, which indicates that they have non-tumorigenic potential and EC-specific characteristics.

### 2.4. Maintenance of TEC-Specific Characteristics in Immortalized ECs

**Figure 4:**
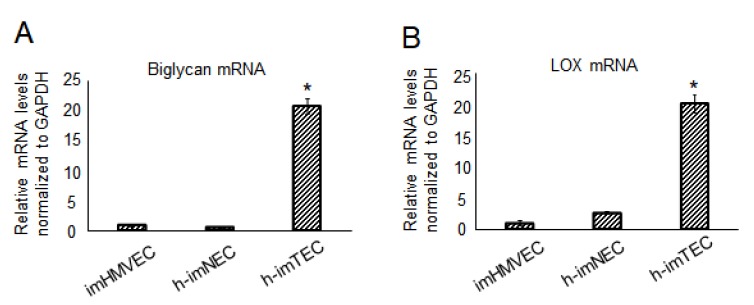
Upregulation of TEC-specific markers in h-imTECs. Biglycan (**A**) and lysyl oxidase (LOX) (**B**) expression was evaluated by real-time PCR (* *p* < 0.01 versus imHMVECand h-imNEC, one-way ANOVA. Data are mean ± SD, *n* = 4 real-time RT-PCR runs). imHMVEC (P12), h-imNEC (P13), and h-imTECs (P9).

We have reported that TECs upregulate specific genes, such as *biglycan (Bgn)* [11,20] and *lysyl oxidase* (*Lox)* [12], compared with NECs. *Bgn* is a small leucine-rich repeat proteoglycan enriched in extracellular matrix. *Bgn* is involved in the mineralization of bone [21]. *Bgn* is upregulated in murine and human TECs of several types of tumors [11,20], and the expression is regulated by DNA methylation [20]. In vitro examination showed that *biglycan* is involved in TEC migration and tube formation [11], and *biglycan* secreted from TECs stimulates tumor cells to metastasize to lungs [20]. *Lox* is a copper-containing amine oxidase and crosslinks collagens and elastins. *Lox*, which is also upregulated in both murine and human TECs, is also involved in tumor angiogenesis [12]. We therefore analyzed the expression of these genes in immortalized ECs. Expectedly, h-imTECs expressed both *Bgn* and *Lox* at high levels compared with NECs, h-imNECs, and imHMVECs (Figure 4A,B and Figure S2A,B).

**Figure 5:**
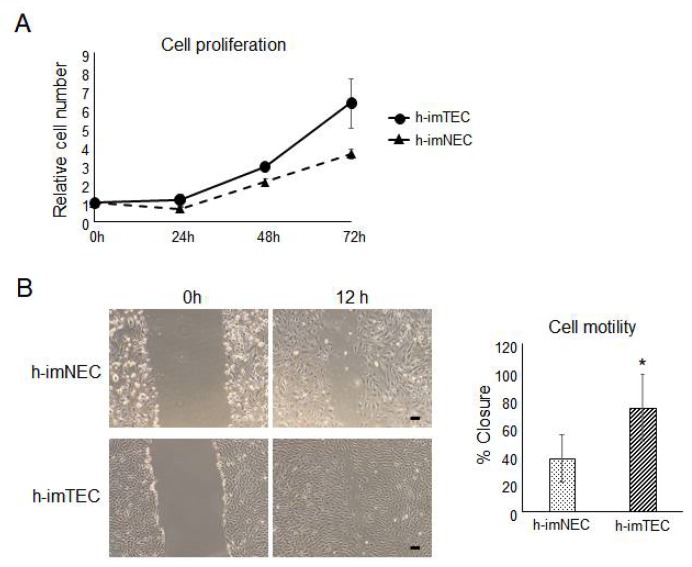
Enhanced proliferation and motility of h-imTECs. (**A**) Cell proliferation assay was performed by cell counting (*n* = 3). h-imNEC (P70) and h-imTECs (P69). (**B**) Cell motility was evaluated by wound healing assay. Representative images (left) and quantitative data (right) were shown. Data is presented as average percent closure ± SD (*n* = 3). h-imNEC (P34) and h-imTECs (P33). (* *p* < 0.05 versus h-imNEC).

Since TECs are known to proliferate faster than NECs [10], we investigated whether h-imTECs showed activated proliferation. By analyzing proliferation by cell counting, it was observed that h-imTECs indeed proliferated faster than h-imNECs (Figure 5A and Figure S3A), which was consistent with the PD of the cells (Figure 2A and Figure S3B). In addition, a motility assay was performed to check differences in migration ability between h-imNECs and h-imTECs. h-imTECs migrated faster than h-imNECs (Figure 5B), which is consistent with our previous report on non-immortalized TECs and NECs [10].

**Figure 6:**
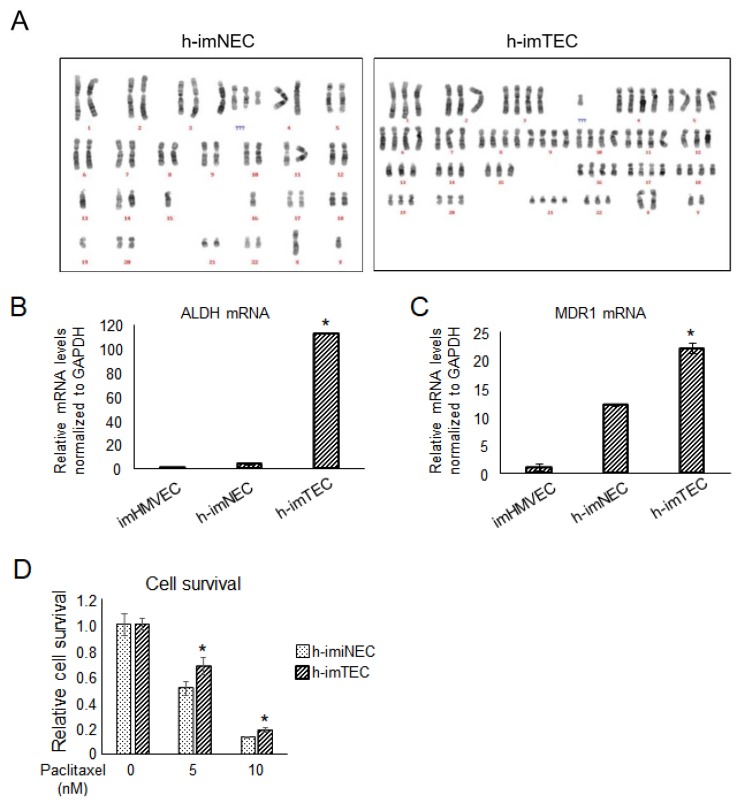
Maintenance of TEC-specific characteristics in h-imTECs. (**A**) Karyotype analysis by Q-banding. h-imNECs (P28) and h-imTECs (P29). (**B**) Aldehyde dehydrogenase (ALDH) expression was evaluated by real-time PCR (* *p* < 0.01 versus imHMVEC and h-imNEC, one-way ANOVA. Data are mean ± SD, *n* = 4 real-time RT-PCR runs). imHMVEC (P26), h-imNEC (P31), and h-imTECs (P9). (**C**) Multidrug resistance 1 (MDR1) expression was evaluated by real-time PCR (* *p* < 0.01 versus imHMVEC and h-imNEC, one-way ANOVA. Data are mean ± SD, *n* = 4 real-time RT-PCR runs). imHMVEC (P35), h-imNEC (P31), and h-imTECs (P30). (**D**) Effect of paclitaxel on EC cell survival was examined by cell counting (*n* = 3). h-imNEC (P25), and h-imTECs (P19). (* *p* < 0.05 versus h-imNEC).

We previously found that TECs have chromosomal abnormalities [6,7,22]. Karyotype analysis by Q-banding demonstrated that h-imTECs had more complex and abnormal karyotypes compared with h-imNECs (Figure 6A). h-imTECs showed several missing chromosomes, an abnormal number of chromosomes, and markers of unknown origin (Figure 6A and Figure S4). In a similar order, old passages of h-imNECs had an abnormal number of chromosomes, suggesting that the long-term culture of h-imNECs will induce chromosomal instability in the cells (Supplementary Figure S4). Aldehyde dehydrogenase (ALDH) is an enzyme that plays an important role in the metabolism of aldehydes. Since several stem cells possess high ALDH activity, ALDH is used as a stem cell marker [23,24]. We previously found that some TECs have high ALDH enzymatic activity [25]. TECs with high ALDH enzymatic activity (ALDH^high^ TECs) sustained their tube formation for longer periods than ALDH^low^ TECs, which suggests that ALDH^high^ TECs may have a relatively higher angiogenic potential. As we previously reported that ALDH^high^ TECs show a higher grade of aneuploidy [17], and ALDH is reported to be involved in chromosomal instability [26], we compared ALDH expression between immortalized ECs. As expected, the expression of ALDH was upregulated in h-imTECs compared with imHMVEC and h-imNECs (Figure 6B). As TECs are known to be resistant to the anticancer drug paclitaxel with the upregulation of MDR1 gene [15,27], we next checked the expression of MDR1 in ECs. As shown in Figure 6C, MDR1 mRNA levels were high in h-imTECs compared with those in imHMVEC and h-imNECs. Consequently, we compared the response of h-imNECs and h-imTECs to paclitaxel. h-imTECs showed more resistance to paclitaxel, which correlates with the MDR1 gene upregulation in the cells (Figure 6D). These data suggested that h-imTECs maintained their TEC-specific characteristics.

**Figure 7:**
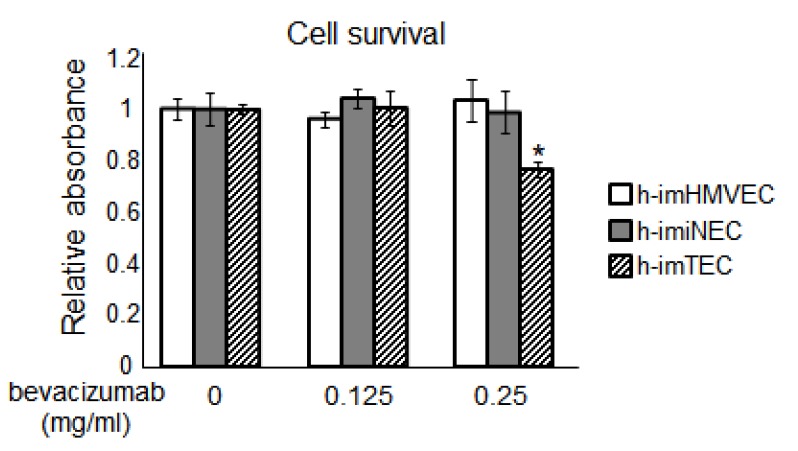
Effect of bevacizumab on EC cell survival as examined by 3-(4,5-dimethylthiazol-2-yl)-5-(3-carboxymethoxyphenyl)-2-(4-sulfophenyl)-2*H*-tetrazolium, inner salt (MTS) assay (* *p* < 0.01 versus 0 mg/mL, one-way ANOVA. Data are mean ± SD, *n* = 4). imHMVEC (P42), h-imNEC (P43), and h-imTECs (P44).

Finally, to examine whether these immortalized ECs can be used for cell-based screening to find novel drug candidates specific for TECs, we treated immortalized ECs with bevacizumab, a well-known antiangiogenic drug, and performed a 3-(4,5-dimethylthiazol-2-yl)-5-(3-carboxymethoxyphenyl)-2-(4-sulfophenyl)-2*H*-tetrazolium,inner salt (MTS) assay. Bevacizumab specifically inhibited h-imTEC cell survival compared with NECs (Figure 7). These results suggested that h-imTECs could be used for chemical cell-based screening with normal controls h-imNECs, and h-imHMVECs. 

## 3. Discussion

In this study, using the ectopic expression of SV40 large T antigen and hTERT, we succeeded in the immortalization of hTECs with their specific characteristics.

Most studies of ECs have been performed using HMVECs or human umbilical vein ECs (HUVECs) isolated from the human normal dermis and umbilical vein, respectively. However, because most human cells including ECs easily undergo cellular senescence after a limited number of cell divisions, researchers frequently need to purchase or isolate ECs from tissue. In addition, the behavior of ECs might vary if the origins of the donors are different. Because of the limitations of primary ECs described above, a number of researchers have immortalized several types of ECs, such as HUVECs [28], HMVECs [29], human liver ECs [30], and human corneal ECs [31].

For the development of new antiangiogenic drugs that efficiently target ECs in tumors without harmful effects on NECs, TECs might be ideal tools. However, current antiangiogenic drugs were developed by researchers using NECs, such as HUVEC, because the isolation of TECs is labor-intensive, and primary human TEC culture is technique-sensitive. In the current study, we cultured h-imTECs with continuous growth and stable TEC properties.

We used SV40 large T antigen and hTERT to immortalize ECs as shown in Figure 1. The overexpression of the gene for SV40 large T antigen has been widely used for replicative senescence. SV40 binds with p53 and pRB, which facilitates cell proliferation. In addition to SV40, most reports use other techniques for immortalization. One of the mechanisms of growth arrest is progressive telomere shortening, which inhibits the cell cycle. Telomerase is the enzyme responsible for maintaining the length of the telomeres by the addition of TTAGGG sequences. In most human somatic cells, telomerase is inactivated or not expressed, and the replication potential becomes limited [32]. This is important to prevent tumorigenesis; however, when developing medicines using high numbers of human normal somatic cells, it is quite difficult to maintain the cells in a phenotypically youthful state. Therefore, the ectopic expression of the *hTERT* gene has also been used as an alternative method for immortalization. However, the induction of hTERT alone is sometimes not enough [33]. Indeed, when we transducted hTERT alone in ECs, their life span was prolonged but not immortalized (data not shown). Accordingly, the co-expression of SV40 large T antigen and hTERT was effective and efficient in immortalizing cells, including ECs. High telomerase activity and unlimited replicative potential are characteristics of germ cells, stem cells, and tumor cells. Among these, germ cells and stem cells behave like normal cells [34,35], implying that increased telomerase activity does not necessarily imply an oncogenic transformation. In fact, the immortalized ECs that we established in this study maintained endothelial characteristics and were not malignantly transformed (Figure 3).

It is known that TECs show specific characteristics, such as upregulation of some specific genes [4,6] and proangiogenic phenotype [10,36]. As shown in Figure 4, our current data indicate that immortalized TECs sustained a high expression of TEC markers including *Bgn* and *Lox*. h-imTECs also demonstrated a proangiogenic phenotype with activated proliferation and migration (Figure 5), which are important for angiogenesis. We previously reported that TECs have chromosomal abnormalities [6,7], and found that hypoxia and accumulation of reactive oxygen species are two of the mechanisms causing chromosomal abnormalities [22]. h-imTECs revealed abnormal and complex chromosomes compared with h-imNECs by karyotype analysis, as shown in Figure 6. Conversely, some reports showed that the transfection of SV40 for immortalization drives karyotypic instability [37]. We were not able to analyze the karyotype in h-NEC and h-TEC due to the limited cell numbers. Whether the slight abnormality observed in h-imNECs was caused by SV40 large T antigen and hTERT transduction needs to be investigated in a further study. In addition, when the karyotype in h-imNECs was analyzed after long-term culture, chromosomal instability was induced compared to the younger passage of h-imNECs. In this study, using the ectopic expression of SV40 large T antigen and hTERT, we succeeded in the long-term culture of both h-imNECs and h-imTECs without senescence, and h-imTECs sustained their specific characteristics compared to normal counterparts. However, some of their characteristics, such as proliferation rate and TEC marker expression might be changed over time, even though the expression of EC markers was maintained in both h-imNECs and h-imTECs. Therefore, these immortalized NECs need to be used in early passages depending on the research purposes.

When immortalized ECs were treated with bevacizumab, an anti-VEGF drug, only TEC proliferation was inhibited (Figure 7), which is consistent with our previous data suggesting that VEGF signaling is more activated in TECs with the upregulation of *VEGF-A*, *VEGFR1*, and *VEGFR2* genes [36,38]. Since the MTS assay, a cell metabolic activity assay, is frequently and widely used to monitor cell proliferation/viability, and is useful for the screening of inhibitors/compounds in cell-based assays, this experimental system was used in the current study. However, the difference in metabolism between the cells requires further analysis.

The availability of the sets of primary human NECs and TECs with immortal lifespan and their specific characteristics will serve as a valuable research tool.

## 4. Materials and Methods

### 4.1. Human Tissue Samples

Tissues from renal tumor clinically diagnosed as RCC were surgically resected. All protocols were approved by the Institutional Ethics Committee of Hokkaido University, and written informed consent was obtained from each patient before surgery. Samples were surgically excised from the tumor tissues and from corresponding normal renal tissues, 5–10 cm apart from the tumor. Tissues were placed in Hanks’ Balanced Salt Solution (Gibco^®^ Thermo Fisher Scientific, Inc., Waltham, MA, USA) on ice until EC isolation. The final diagnosis of RCC was confirmed by pathologic examination of formalin-fixed surgical specimens.

### 4.2. Isolation of hTECs and hNECs and Cell Culture

Excised human RCC tissue and normal kidney tissue were processed by a magnetic cell separation system (MACS; Miltenyi Biotec, Bergisch Gladbach, Germany) to isolate hTECs and hNECs as described previously [7] with some modifications. Briefly, excised tissues were minced and digested with collagenase II (Gibco^®^ Thermo Fisher Scientific, Inc., Waltham, MA, USA) for 1 h at 37 °C. Blood cells were removed by lysing buffer (BD Biosciences, San Jose, CA, USA). The cells were then incubated with FcR blocking reagent (Miltenyi Biotec, Bergisch Gladbach, Germany) followed by CD31 microbeads (Miltenyi Biotec, Bergisch Gladbach, Germany). hTECs and hNECs were isolated by a MACS according to the manufacturer’s instructions. To improve the purity of hTECs and hNECs, cells were isolated as a CD31^+^CD45^−^ population with Alexa Fluor 647-conjugated anti-human CD31 antibody (Biolegend, San Diego, CA, USA) and phycoerythrin-conjugated anti-human CD45 antibody (Biolegend, San Diego, CA, USA) by the FACSAria II (BD Biosciences, San Jose, CA, USA). They were plated on 1.5% gelatin (Sigma-Aldrich, St Louis, MO, USA) and 10 µg/mL fibronectin (Corning, Corning, NY, USA)-coated dishes, and cultured in EGM-2MV (Microvascular Endothelial Cell Growth Medium-2, Lonza, Basel, Switzerland) with additional 15% heat-inactivated fetal bovine serum (FBS). Human microvascular ECs (HMVECs) were purchased from Lonza (Basel, Switzerland) and cultured in EGM-2MV. Both MS1 cells, murine ECs immortalized by sequential introduction of SV40 large T antigen, which were purchased from the American Type Culture Collection (ATCC, Manassas, VA, USA), and 293T cells, purchased from RIKEN Cell Bank (Tsukuba, Japan) were cultured in Dulbecco’s Modified Eagle Medium (DMEM) with 10% FBS. A human melanoma cell line, A375SM, was kindly supplied by Dr. Isaiah J. Fidler (MD Anderson Cancer Center). These cells were cultured in minimum essential medium (Gibco^®^ Thermo Fisher Scientific, Inc., Waltham, MA, USA) with 10% FBS. All cells were cultured at 37 °C in a humidified atmosphere containing 5% CO_2_. The absence of *Mycoplasma pulmonis* was checked by polymerase chain reaction (PCR).

### 4.3. Plasmids and Transfection

pCSII-CMV-hTERT-IRES2-Venus was previously generated and published [39]. pRRLsin-SV40 T antigen-IRES-mCherry was purchased from Addgene (Cambridge, MA, USA). Lentiviruses were produced using 293T cells co-transfected with packaging constructs pCAG-HIVgp and the VSV-G- and REV-expressing construct pCMV-VSV-G-RSV-REV (from H. Miyoshi), pCSII-CMV-hTERT-IRES2-Venus, or pRRLsin-SV40 T antigen-IRES-mCherry. Infection was achieved as reported previously [20]. To purify transducted cells, each EC type was analyzed and sorted by flow cytometry (FACSAria II) with Venus and mCherry signals. Venus, mCherry, and CD31-positive cells were sorted after sub-culture as immortalized ECs. Data were analyzed using FlowJo software (Tree Star, Inc., Ashland, OR, USA).

### 4.4. Isolation of RNA, Reverse Transcription-PCR (RT-PCR), and Quantitative PCR

Total RNA was isolated using the ReliaPrep™ RNA Cell Miniprep System (Promega, Madison, WI, USA) according to the manufacturer’s instructions. cDNA was synthesized using ReverTra-Plus (Toyobo, Osaka, Japan) and amplified by PCR. PCR products were visualized by ethidium bromide staining. Quantitative real-time RT-PCR was performed using the KAPA SYBR^®^ FAST qPCR Kit (KAPA Biosystems, Boston, MA, USA) according to the manufacturer’s instructions. Cycling conditions were set based on CFX Manager (Bio-Rad, Hercules, CA, USA). mRNA expression levels were normalized to that of glyceraldehyde 3-phosphate dehydrogenase (GAPDH) and analyzed using the Δ-Δ-Ct method. The primers used were as follows: SV40 forward 5′-GGTGGGTTAAAGGAGCATGA-3′ and reverse 5′-CAACTCCAGCCATCCATTCT-3′, hTERT forward 5′-GCATCAGGGGCAAGTCCTAC-3′ and reverse 5′-CCAACAAGAAATCATCCACCAA-3′, mouse *Gapdh* forward 5′-CACTGAGCATCTCCCTCACA-3 and reverse 5′-GTGGGTGCAGCGAACTTTAT-3′, human *Gapdh* forward 5′-ACAGTCAGCCGCATCTTCTT-3′ and reverse 5′-GCCCAATACGACCAAATCC-3′, human *CD31* forward 5′-GACCAGGTGAAAGACTGAACC-3′ and reverse 5′-TGCAGATATACGTCCCACTGTC-3′, human *CD144* forward 5′-CGTGTTCGCCATTGAGAGGC-3′ and reverse 5′-ACGGACGCATTGAACAACCG-3′, human *biglycan (bgn)* forward 5′-GGCCATCCATCCAGTTTGGCAACTAC-3′ and reverse 5′-CTGGCTTAGCTTCCTGGCTCTG-3′, and human *lysyl oxidase* (*Lox*) forward 5′-CGACCCTTACAACCCCTACA-3′ and reverse 5′-CAGGTCTGGGCCTTTCATAA-3′.

### 4.5. Determination of Population Doubling (PD) Time

Each EC was seeded at 5.0 × 10^3^ cells per well on 24-well plates. After 4 days of culture, the cells were harvested and counted. The cells did not reach confluency during the culture in this condition. The PD level was calculated for each subculture using the following equation:

PD time = log (final cell number) − log (initial cell number)

The calculated PD increase was added to the PD levels of the previous passages to yield the cumulative PD level.

### 4.6. Senescence-Associated β-Galactosidase (SA-β-Gal)

Cells were expanded upon reaching sub-confluency. SA-β-Gal activity was analyzed using the Senescence Detection Kit (Abcam, Cambridge, UK) according to the manufacturer’s instructions. Briefly, cells were washed with phosphate-buffered saline and fixed with fixative solution followed by staining with the X-Gal-containing staining solution mix at 37 °C. Positive cells had cytoplasmic staining that appeared blue.

### 4.7. Tube Formation Assay

Growth factor-reduced Matrigel (Corning, Corning, NY, USA) was placed in each well of a 96-well dish and incubated at 37 °C for 30 min. Cells were seeded at 1.0 × 10^4^ per well in EBM-2 (Lonza, Basel, Switzerland) containing 5% FBS, and incubated at 37 °C for 9 h. Tube formation was observed using an inverted microscope (CKX41, Olympus, Tokyo, Japan).

### 4.8. Soft Agar Colony Formation Assay

Cells were harvested, gently mixed with 0.36% agar medium mixture (Difco Agar Noble, BD Biosciences, San Jose, CA, USA), and re-seeded on six-well plates covered with 0.9% agar in EGM-2MV. After one week, the colonies were observed using an inverted microscope (Olympus, Tokyo, Japan).

### 4.9. Karyotype Analysis

The Q-banding technique was used for karyotype analysis by Chromosome Science Labo, Inc. (Sapporo, Japan), as described previously [22]. Fifty karyotypes of each ECs were analyzed.

### 4.10. Cell Survival Assay

To examine the effect of paclitaxel, ECs were seeded at 1 × 10^4^ per well in EGM-2MV and incubated at 37 °C for 24 h before adding paclitaxel. After 48 h and 72 h, cell survival was measured by cell counting. To examine the effect of bevacizumab (Avastin), ECs were seeded at 1 × 10^3^ per well in EBM-2 containing 5% FBS, and incubated at 37 °C for 24 h before adding bevacizumab. After 48 h, cell survival was measured by MTS assay (Promega, Tokyo, Japan). VEGF concentration was as supplied by the manufacturer of the commercial medium.

### 4.11. Wound Healing Assay

Cells (1 × 10^5^) were plated on 12-well plates in EGM-2MV and allowed to create a confluent monolayer. The monolayer was scratched with a P200 pipette tip. Image acquisition was performed immediately after scratching and at 12 h after scratching. Images were obtained using an inverted microscope (Olympus, Tokyo, Japan) and analyzed using ImageJ software (NIH, Bethesda, MD, USA).

### 4.12. Statistics

All data are expressed as mean ± standard deviation (SD) of three independent experiments performed in triplicate, and subjected to one-way analysis of variance (ANOVA) followed by a Tukey–Kramer’s multiple comparison test. A two-sided Student’s *t* test was used for comparison between two groups. *p* < 0.01 was considered statistically significant. 

## Supplementary Materials

The following are available online at https://www.mdpi.com/1422-0067/20/18/4595/s1.

## Abbreviations

ALDH	aldehyde dehydrogenase
ATCC	American Type Culture Collection
BGN	bigycan
EC	endothelial cell
FBS	fetal bovine serum
GAPDH	glyceraldehyde 3-phosphate dehydrogenase
h-imTEC	immortalized human tumor endothelial cell
h-imNEC	immortalized human normal endothelial cell
HMVEC	human microvascular endothelial cell
hNEC	human normal endothelial cell
hTEC	human tumor endothelial cell
hTERT	human telomerase reverse transcriptase
HUVEC	human umbilical vein endothelial cell
imHMVEC	immortalized HMVEC
LOX	lysyl oxidase
MDR1	multidrug resistance 1
MTS	3-(4,5-dimethylthiazol-2-yl)-5-(3-carboxymethoxyphenyl)-2-(4-sulfophenyl)-2*H*-tetrazolium,inner salt
NEC	normal endothelial cell
PD	population doubling
RCC	renal cell carcinoma
RT-PCR	reverse transcription-PCR
SA-β-Gal	Senescence-associated β-galactosidase
SV40	simian virus 40
TEC	tumor endothelial cell
