# Supplementary material for: Development of Immortalized Human Tumor Endothelial Cells from Renal Cancer
Source: Int J Mol Sci. 2019 Sep 17;20(18):4595. doi: 10.3390/ijms20184595 (PMC6770423; doi:10.3390/ijms20184595)
Supplement: Supplementary file 1 [file ijms-20-04595-s001.pdf]

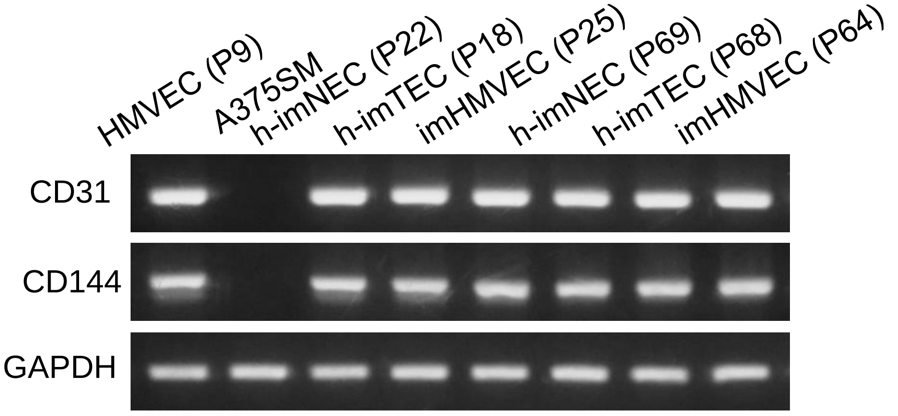

Figure S1. EC marker, CD31, and CD144 mRNA expression levels in each EC at different passages were determined by RT-PCR. HMVEC and A375SM were used as positive and negative controls, respectively.

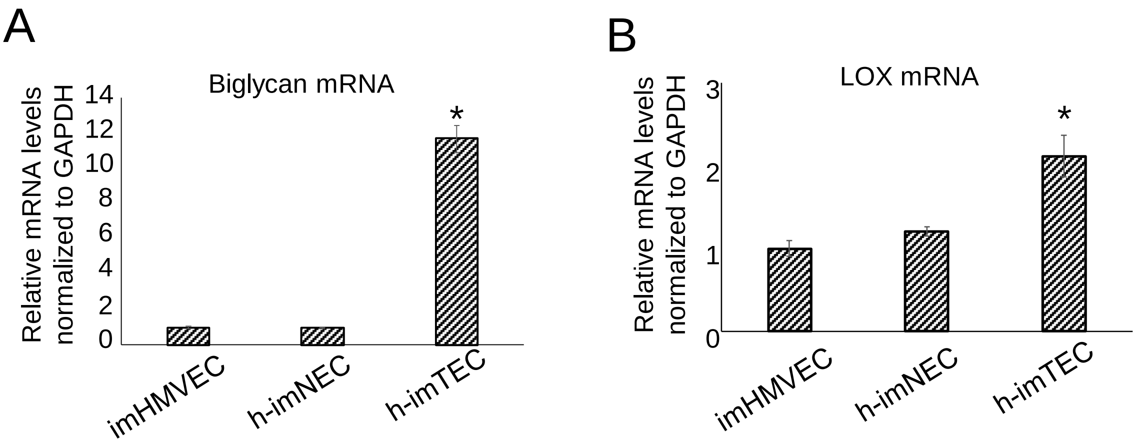

Figure S2. Upregulation of TEC-specific markers in h-imTECs. Biglycan (A) and LOX (B) expression was evaluated by real-time PCR (*$P < 0.01$ versus imHMVEC and h-imNEC, one-way ANOVA. Data are mean ± SD, n = 4 real-time RT-PCR runs). imHMVEC (P36), h-imNEC (P37), and h-imTECs (P36).

A

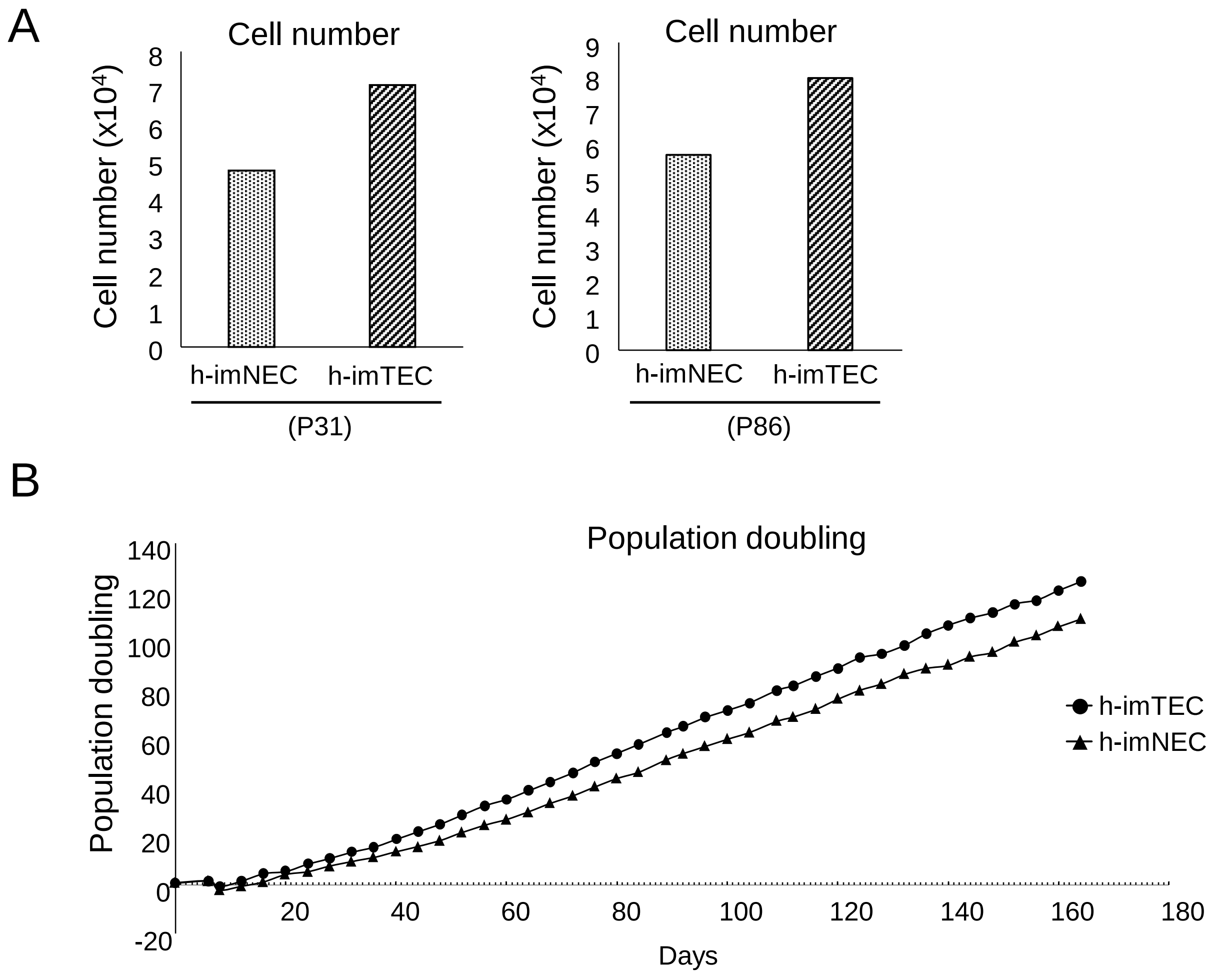

Figure S3. Enhanced proliferation of h-imTECs. (A) Cell proliferation was assessed by cell counting after incubation for 96h between h-imNEC and h-imTECs in P31 (left) and P86 (right), respectivbely. (B) Growth kinetics of each EC was measured by cell counting, and PD was calculated. This is the merged data of Figure 2A.

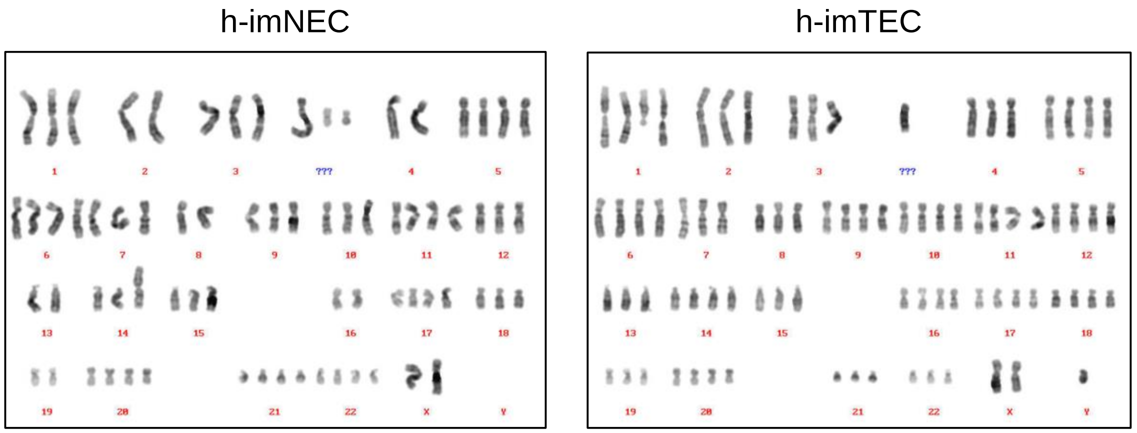

Figure S4. Karyotype analysis by Q-banding. h-imNECs (P67) and h-imTECs (P66).
